# Purinergic Signaling and Cochlear Injury-Targeting the Immune System?

**DOI:** 10.3390/ijms20122979

**Published:** 2019-06-18

**Authors:** László Köles, Judit Szepesy, Eszter Berekméri, Tibor Zelles

**Affiliations:** 1 Department of Pharmacology and Pharmacotherapy, Semmelweis University, H-1089 Budapest, Hungary; koles.laszlo@med.semmelweis-univ.hu (L.K.); szepesy.judit@med.semmelweis-univ.hu (J.S.); berekmeri.eszter@gmail.com (E.B.); 2 Department of Ecology, University of Veterinary Medicine, H-1078 Budapest, Hungary; 3 Department of Pharmacology, Institute of Experimental Medicine, Hungarian Academy of Sciences, H-1083 Budapest, Hungary

**Keywords:** purinergic signaling, inflammation, immune response, organ of Corti, sensorineural hearing losses, noise-induced hearing loss, drug-induced hearing loss, age-related hearing loss

## Abstract

Hearing impairment is the most common sensory deficit, affecting more than 400 million people worldwide. Sensorineural hearing losses currently lack any specific or efficient pharmacotherapy largely due to the insufficient knowledge of the pathomechanism. Purinergic signaling plays a substantial role in cochlear (patho)physiology. P2 (ionotropic P2X and the metabotropic P2Y) as well as adenosine receptors expressed on cochlear sensory and non-sensory cells are involved mostly in protective mechanisms of the cochlea. They are implicated in the sensitivity adjustment of the receptor cells by a K^+^ shunt and can attenuate the cochlear amplification by modifying cochlear micromechanics. Cochlear blood flow is also regulated by purines. Here, we propose to comprehend this field with the purine-immune interactions in the cochlea. The role of harmful immune mechanisms in sensorineural hearing losses has been emerging in the horizon of cochlear pathologies. In addition to decreasing hearing sensitivity and increasing cochlear blood supply, influencing the immune system can be the additional avenue for pharmacological targeting of purinergic signaling in the cochlea. Elucidating this complexity of purinergic effects on cochlear functions is necessary and it can result in development of new therapeutic approaches in hearing disabilities, especially in the noise-induced ones.

## 1. Introduction—the Hearing Organ

The hearing organ of the mammals is located in the ossified labyrinth of the temporal bone. Inside the bony capsule the membranous labyrinth contains the organ of Corti spiralled up around the modiolus. The whole structure is shaped like a snail shell and it has been designated as cochlea (from an Ancient Greek word). Two membranes (basilar and Reissner’s membrane) divide the spiralling canal into three different compartments (scalae). Scala vestibuli (the upper) and scala tympani (the lower compartment) contain perilymph, scala media (middle compartment) is filled with endolymph. Perilymph has an ion composition similar to that of the cerebrospinal fluid [1,2,3] while endolymph has high K^+^ and low Na^+^ levels [1,2,3]. Consequently, the endolymph is 80–100 mV more positive than the perilymph (endocochlear potential), furthermore, 125–150 mV more positive than hair cells interior at their resting potential [1]. The electrochemical gradient generates ion-flow (mostly K^+^) into these receptor cells resulting in depolarization upon opening of the mechanoelectric transduction channels. These non-selective cation channels are located on the top of the stereocilia on hair cells and are activated in response to the noise-evoked tilting of the hair bundle [1,2,4].

**Figure 1:**
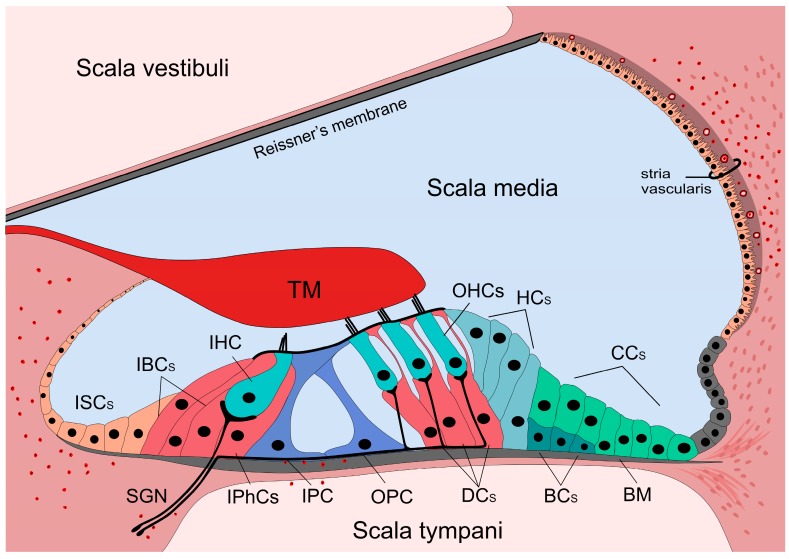
Anatomy of the organ of Corti and the presence of immune cells. The cochlea is divided into three chambers (scalae) by two membranes. The organ of Corti is located in the scala media. The three rows of outer hair cells (OHCs) and the one row of inner hair cells (IHCs) are surrounded by different types of supporting cells. The cells are surrounded by the perilymph, but the stereocilia of the hair cells are bathed in the endolymph. The reticular lamina is formed by the apical parts of the cells establishing a barrier between the endo- and perilymphatic fluid compartments. The basilar membrane (BM) separates the scala media and tympani. Supporting cells (IBCs, IPhCs, IPC, OPC, DCs, HCs, CCs, BCs) span through the cellular layer of the organ while hair cells (IHC and OHCs) are not in direct contact with the BM, but their stereocilia reaches the tectorial membrane (TM). Resident macrophages and leukocytes are always present in the cochlea, primarily in the spiral limbus, in the scala tympani side of the BM as well as in the lateral wall. Red formations with black dots in the middle show position of these immune cells. ISCs: Inner sulcus cells; IBCs: Inner boarder cells; IPhCs: Inner phalangeal cells; IPC: Inner pillar cell; OPC: Outer pillar cell; DCs: Deiters’ cells; HCs: Hensen’s cells; CCs: Claudius’ cells; BCs: Boettcher’s cells. IHC and OHCs: Inner and outer hair cells; SGNs: Spiral ganglion neurons; TM: Tectorial membrane.

The organ of Corti is situated on the basilar membrane (BM). It is composed of sensory (inner and outer hair) cells and supporting cells. The cell bodies are bathed in the perilymph, but their apical parts are connected with tight junctions forming the reticular lamina, an efficient barrier between the peri- and endolymph (Figure 1).

Hair cells don’t contact directly to the BM, but their stereocilia extend to the endolymph. The three rows of the outer hair cells (OHCs) are supported by the three rows of Deiters’ cells (DCs), and the single row of inner hair cells (IHCs) is surrounded by the inner border and inner phalangeal cells. In the middle of the organ, inner and outer pillar cells form the tunnel of Corti.

The medial part next to the IHCs and their supporting cells is followed by the spiral limbus cuboidal cells. Above the spiral limbus the tectorial membrane (TM) overlies the hair cells, deflects their stereocilia of the OHCs thereby depolarizing or hyperpolarizing them. Lateral to the DCs the cylindrical Hensen’s cells (HCs), the cuboidal Claudius’ cells (CCs) and the Boettcher’s cells (BCs) (in the basal turn of the cochlea) close the organ.

Although CCs and the BCs are often not considered as the part of the classical organ of Corti, many researchers have an opposing view as they are also involved in one of the most important function denoted to supporting cells, namely the K^+^ spatial buffering pathway.

The lateral wall is formed by the spiral ligament containing a vascular part (stria vascularis) covered by the marginal cells. It produces the endolymph [1,5,6]. Special characteristics of strial microvessels are responsible for the blood-labyrinth barrier (BLB), an effective barrier between the endolymph and the blood [7,8].

Hair cells are innervated by the spiral ganglion neurons (SGNs). The cell body of these bipolar neurons is located in the modiolus of the cochlea and the central processes join to the nerves from the vestibular part and form the 8th cranial (vestibulocochlear) nerve. SGNs are divided into two subtypes. Type I SGNs innervate IHCs, one neuron innervates only one IHC and one IHC gets about 10 innervations [9,10]. Type II SGNs innervate more OHCs, and one OHC gets only one innervation [11,12].

Previously, the BLB was proposed to be an immune barrier and so the cochlea was thought to be isolated from the systemic immune system [7]. On the contrary, more recently resident macrophages were found in several parts of the cochlea (e.g., spiral limbus, BM, cochlear lateral wall) even without any damage of the BLB [13,14]. In response to injury (e.g., noise exposure, ischemia) they are activated and consequently the immune response will be increased [15,16,17]. Inflammatory cytokines released by these macrophages and macrophage-like melanocytes can increase BLB permeability [16,17], and BLB can be disrupted in case of cochlear insults such as inflammation, noise, age or ototoxic drugs [18,19]. Immune cells can invade from the periphery and escalate the immune response [20]. Cochlear immune-responses may involve even the ability of supporting cells to clear apoptotic cells in their neighbourhood by phagocytosis [21].

This review focuses on two peculiar fields: (i) The role of purinergic signaling in cochlear physiology and pathology and (ii) the description of cochlear immune system and its involvement in cochlear damage as well as their interaction, namely the role of purinergic signaling in immune system regulation in the hearing organ. We provide an overview of the purinergic signaling in the cochlea, depicting its components and P2 receptor-mediated mechanisms influencing the cochlear functions such as K^+^ recycling, cochlear amplification and membrane rigidity, intercellular Ca^2+^ waves or development of the organ of Corti. Adenosine receptor-mediated mechanisms influencing the cochlear functions will also be described briefly. The following two sections resume the sensorineural hearing losses (SNHLs) and discuss the role of the purinergic signaling in its pathology. Overview of the purinergic modulation of the immune system and the involvement of the immune mechanisms in SNHL pathology at the end of this review draw the reader’s attention to the possibility of SNHL therapy via purinergic regulation of the cochlear immune response.

## 2. Purinergic Signaling and its Components in the Cochlea

In addition to the fundamental intracellular role of ATP as universal energy source, nucleotides and nucleosides fulfil crucial extracellular roles as messenger molecules and modulators [22,23,24].

Virtually all cell types can be the source of extracellular nucleotides releasing ATP (and UTP) into the extracellular space by synaptic and extrasynaptic vesicular release, channel-mediated release (e.g., connexin gap junction hemichannels, non-junctional pannexin channels, plasmalemmal voltage-dependent anion channels, osmotic transporters linked to anion channels and even P2X7 receptors operating as ATP-permeable channels) [24,25,26]. ATP also appears extracellularly upon cell breakdown exposing cells to high concentrations of purines after cell death in the neighbouring areas [27]. It can result in protective effects but it can also escalate detrimental mechanisms thereby intensifying destruction and damage [28,29,30].

Nucleotides in the extracellular space are rapidly hydrolyzed and interconverted by ectoenzymes (ecto-ATPases, ecto-apyrases and ectonucleotidases) resulting in active metabolites (e.g., ADP, adenosine) with altered receptor selectivity or terminating their signaling action [31]. Since receptors for adenosine (P1) and ATP/ADP (P2 receptors) are usually functionally antagonistic, extracellular metabolism of ATP–besides limiting its actions by enhancing its removal–brings also new players, with a new activity/modulatory profile into the game, as well. As a result, extracellularly, in virtually all tissues, we have a complex purinergic regulatory system with the involvement of P2 receptors (further subdivided into numerous subtypes–each of them fulfil distinctive roles), nucleotide metabolizing (hydrolyzing and interconverting) enzymes, and the P1 (i.e., adenosine) receptors [24,25].

P2 receptors are among the most abundant receptors: They are expressed in all types of cells in mammalian tissues. They have been divided into two types: Ligand-gated cation channels (P2X) and G protein-coupled receptors (P2Y) [32]. Seven different P2X subunits (P2X1–7) can co-assemble in homo- or heterotrimers to form a channel and eight distinct P2Y subunits (P2Y1, P2Y2, P2Y4, P2Y6, P2Y11–14) in homo- or hetero-oligomeric complexes couple mostly to G_s_, G_q/11_ and/or G_i/o_ proteins (P2Y1, P2Y2, P2Y4, P2Y6 and P2Y11 mostly to G_q_, while P2Y12–14 predominantly to G_i_) [33,34]. P2Y1, P2Y11, P2Y12, and P2Y13 receptors are activated by purine ligands (ATP or ADP), while P2Y4, P2Y6, and P2Y14 are pyrimidine-selective (i.e., activated by UTP/UDP). P2Y2 receptor is activated by both ATP and UTP [33,35].

Adenosine (P1 or A) receptors are divided into four major subtypes: A1, A2A, A2B and A3. A1 and A3 receptors are coupled to G_i/o_ protein, while both A2A and A2B are positively coupled to adenylate cyclase. Adenosine is less potent at A2B receptors and they are considered as low-affinity adenosine receptors [36].

Purinergic signaling has an impact on virtually all body functions, influencing e.g., cardiovascular, central nervous system, respiratory, gastrointestinal and genitourinary activities. Indeed, purinergic transmission and modulation has been proposed to be involved in the sensorineural and other auditory functions as well [37,38].

In addition to acting physiologically as a neurotransmitter and/or a neuromodulator, pathologically high concentrations of ATP can also lead up machinery of harmful events thereby intensifying also cochlear destruction and damage [30,39].

Most purinergic receptors have been detected in the organ of Corti, their expression pattern can show age and species dependent distribution (Table 1 and Table 2). Apparently, P2X2 subtype is one of the major purinergic receptors in the hearing organ, being expressed both in sensory cells [10,37,40,41,42,43,44,45,46,47,48,49,50,51,52,53] and also in non-sensory, i.e., supporting cells [10,37,41,47,48,49,50,51,52,53,54,55,56,57,58,59]) in different species and in various ages. The apparent importance of this purinergic receptor in cochlear physiology was highlighted by recent observations demonstrating that mutation of the P2X2 receptor resulted in progressive hearing loss [42]. Other P2X receptors such as P2X1, P2X3 and P2X4 show more restricted, often strong age-dependent expression profile [40,58,60,61]. The P2X7 subtype, supposed to be involved mostly in pathological events as it is typically activated by higher concentration of ATP, is also expressed in the cochlea: Both in sensory cells [40,43,44,62,63] and in non-sensory cells [55,56,62]. Noteworthy is the distinct role of this purinergic receptor subtype in immune functions, and its predominant localisation in immune cells of the body [64].

Among the metabotropic P2Y receptors [40,44,66,67,68,69,70], the P2Y4 subtype shows a widespread expression in all developmental stages, both in sensory [44,66,68,70] and in non-sensory cells [66,67,68,69,70]. Other P2Y subtypes detected in the cochlea are P2Y1, P2Y2, P2Y6 (e.g., rat and guinea pig OHC) [40,66,68] and P2Y12 (e.g., rat OHC) [66,68]. Functional data support the view that P2Y receptor subtypes are also present in supporting cells around hair cells [67,83,84,85]. Pillar cells express (besides P2Y4) P2Y2 and P2Y12 during development [66,67], later additionally P2Y6 (but not P2Y12 anymore) [66]. P2Y2 subtypes are also present in DCs and HCs before hearing onset, but not after that [66,67], in contrast outer sulcus cells, which cell types express them during and after maturation [66,67]. Outer sulcus cells also express P2Y1 after the hearing onset in rat [66]. For details see Table 1.

A1, A2A A2B and A3 adenosine receptor subtypes are also expressed in the hearing organ, after maturation: Both in sensory cells and in SGNs [71,72,73,74,75,76] as well as in non-sensory cells [72,73,76]. High affinity adenosine receptor subtypes show distinct localization in the cochlea (e.g., A1–DCs and IHCs [71,72,73]; A2A–blood vessels and ganglionic neurons [72,73,74,76]; A3-all types of supporting cells, hair cells, inner and outer sulcus cells, spiral ganglia [72,73] (Table 2).

ATP concentration in the extracellular environment is controlled by ectonucleotidases. These enzymes are also present in cochlea of hearing rodents, including their major representatives such as ectonucleoside triphosphate diphosphohydrolase 1 (NTPDase1) and NTPDase3, known to terminate P2 receptor-mediated signal transmission (by hydrolyzing nucleotides), and NTPDase2 preferentially interconverting nucleotides (i.e., producing ADP from released ATP) [77,78,80,81]. NTPDase5 and NTPDase6, preferentially hydrolyzing nucleoside 5’-diphosphates, such as UDP and GDP, are present both in developing and mature hearing organ in rodents [68,82] (Table 3).

## 3. P2 Receptor-Mediated Mechanisms Influencing the Cochlear Functions

Purinergic signaling in physiological conditions confers mostly protective adaptation mechanism on the cochlea. Elevation of ATP level and activation of the purinergic signaling in response to noise stimuli desensitizes and thereby protects the system [38]. However, as we will outline below, the purinergic system can be also involved in deleterious mechanisms threatening the cochlear integrity especially via escalation of the immune responses. It can also explain the controversial reports about the purinergic effects on cochlear functions (see below). Here we depict the major elements of the purinergic (mostly protective) effects in physiological situations or in the case of mild or temporary insults. Several purinergic receptors are involved in these actions, but P2X2 receptor seems to be the major subtype in physiologic conditions (see above).

### 3.1. K^+^ Recycling

Endocochlear potential (see above) is the external driving force for depolarizing K^+^ flow resulting in hair cell activation if deflection of the stereocilia opens the mechanoelectrical transduction channels [4,6,55,86,87]. K^+^ is recycled to the stria vascularis (SV) for secretion back into scala media and thereby maintenance of the endocochlear potential. It occurs in part through the perilymph (by voltage gated K^+^ channels located in the lateral membranes of the cell) and/or utilizing intracellular pathways: Via selective ion channels and gap junctions of supporting cells and further cell types of the epithelial lining (e.g., CCs) [4,44,55]. Finally, K^+^ flows to the lateral wall and SV can resecrete it to the endolymph [6,55].

ATP can mediate this potassium recycling. Namely P2X (mostly P2X2) channels of the cells lining the endolymphatic compartment are permeable to K^+^. In physiological conditions ATP concentration in the endo- and perilymph is in the low nanomolar range, and in response to pathological insults (e.g., noise, hypoxia, ischemia) it can be increased and reach micromolar levels [87]. As a result, K^+^ entry into the cells can be increased, and consequently K^+^ concentration reduced in the endolymph. It can diminish the endocochlear potential and adjust hearing sensitivity [4,41,48,88,89]. Additionally, ATP can mitigate mechanisms involved in the K^+^ recycling: Namely disconnect the gap junction coupling between the supporting cells and inhibit voltage gated channels. It can contribute to K^+^-sinking [90,91,92,93].

Additional factors may further facilitate the protective effect of ATP (decrease in the hearing sensitivity). High proton concentrations have been registered in ischemia or noise induced trauma. Protons can modulate ion flux through P2X channels, e.g., P2X1 currents are inhibited, P2X2 are potentiated while P2X3 currents are reduced at low agonist concentrations and increased at high agonist concentrations in acidic pH [94]. Indeed, ATP induced ion currents at pH 6.5 have been virtually duplicated in isolated DCs [95]. Furthermore, purinergic receptors can be both up- and down-regulated. P2X2 subunit expression has been elevated in cochlear sensory and non-sensory cells in rodents in response to chronic noise exposure [10,96].

### 3.2. Cochlear Amplification and Membrane Rigidity

Cochlear (mechanical) amplification of acoustic signals decreases the hearing threshold by about 40 dB and improves frequency selectivity in the mammalian organ of Corti [97,98,99]. Prestin, a membrane protein, located in the lateral wall of the OHCs is a key factor in cochlear amplification. Prestin-as a piezo crystal-converts the electricity into motion in OHCs, which in turn enforces the activation of the IHCs whose threshold is higher than that of the OHCs [92,100,101].

ATP induced cytoskeleton reorganization in PC12 cells [102]. Both P2X and P2Y receptor subtypes can be involved in actin filament rebuilding. By influencing cell morphology and rigidity, purinergic receptors may affect the cochlear amplification. Contraction of the phalangeal process can directly modulate DCs-OHC mechanical coupling and OHC electromotility. In acute cell culture, ATP elicited movement of phalangeal processes of isolated DCs [103] and also evoked an inward current in supporting cells (see the ATP-induced K^+^-sinking above), thereby possibly regulating the OHC electromotility via the DCs [92]. However, in acute hemicochlea preparations, we failed to demonstrate this phenomenon [104]. Probably the motion of the cellular compartments could not be visualized due to the strong coupling between the cells.

### 3.3. Intercellular Ca^2+^ Waves

Calcium waves can synchronize cells across long distances and can be involved in patho(physiological) events. Indeed, hair cell damage elicited intracellular Ca^2+^ elevations in the neighbouring supporting cells evoking Ca^2+^ waves spreading to several 10–100 µm from the injured area in immature cochlea [67,105,106,107]. ATP set free from the injured cells can induce these increases in intracellular Ca^2+^ levels [104,108]. The propagation of waves may involve two mechanisms: (a) ATP release via hemichannels and activation of ionotropic (i.e., P2X2 and/or P2X4) and metabotropic (i.e., P2Y2) purinergic receptors of neighbouring cells resulted in a faster wave and (b) IP_3_ flow through gap junctions releasing internal Ca^2+^ stores of the neighbouring cells is resulted in a slower wave [43,67,106,109,110,111,112,113,114]. More recently, this phenomenon was also observed in adult mouse organ of Corti [115]. These waves may function as early detectors of cochlear injury, but may also be involved in physiological mechanisms, for instance K^+^ recycling through the supporting cells. Cell regeneration, apoptosis or synaptic maintenance during development and maturation may also utilize this process [116,117].

### 3.4. Development of the Organ of Corti

Purinergic receptors show a characteristic and continuously changing expression pattern until the end of the maturation (the second postnatal week–see Table 1) [10,37,56,58,61,62,65,66,70,118]. ATP, secreted into different compartments of the immature cochlea, can regulate the synapse maturation in opposing ways: In the endolymphatic space by activating hair cells strengthening the synapses, but in the perilymphatic compartment acting on the purinergic receptors expressed on SGNs possibly eliminating (the weaker) synapses.

Supporting cells of the Kölliker’s organ (a transient anatomical structure in the immature cochlea) are thought to induce firing spontaneous action potentials [83,84,119,120,121,122] and this spontaneous activity, observed in the whole auditory pathway, reaches the highest frequency during synapse maturation (around P10–12) [9,83,84,119,123]. Interestingly, the purinergic receptors show also the highest level of expression to this time [47]. The supporting cells release ATP spontaneously and rhythmically, and ATP acts also as a paracrine mediator molecule on neighbouring (supporting and hair) cells by activating their purinergic receptors [43,63,67,83,110,112,124,125,126]. It is resulted in ATP-mediated synchronized depolarization of these cells, and coordination of primary auditory neuron firing is crucially involved in the proper organization (tonotopy) and development of the auditory system [119,127]. For instance, depolarization caused by ATP evokes glutamate release from hair cells and the consequent activation of the SGNs promotes their survival and the maturation of the hair cell-primary auditory neuron synapses [65,83,85,128,129,130,131]. SGNs also express purinergic receptors (primarily P2X2, P2X3, P2X2/3 and also P2Y subtypes) [10,41,52,56,57,58,59,60,66].

Growth factors released by the active synapses are known to influence neurite development [57,132,133,134]. Activation of P2X2 receptors reduced the expression of the brain derived neurotrophic factor (BDNF) and consequently the BDNF-induced neurite outgrowth in cultured SGN neurons [57].

## 4. Adenosine Receptor-Mediated Mechanisms Influencing the Cochlear Functions

Based on the characteristic expression pattern of the high affinity adenosine receptors (A1, A2A and A3–see above), adenosine is implicated in the modulation of sound detection and hearing sensitivity [72]. The G_i/o_-coupled A1 and A3 receptors may control transmitter release (e.g., glutamate from the IHCs) and inhibit neuronal excitability and synaptic transmission in neuronal elements such as spiral ganglia. The predominantly G_s_-coupled A2A receptors may have facilitatory role on the release of excitatory neurotransmitters [135]. Nevertheless, the explicit functional role of adenosine in the cochlear physiology has not yet been clarified [72]. Adenosine is also involved in the regulation of cochlear blood flow (CBF). For instance administration of adenosine into the perilymph increased the CBF [136]. It can be one of the underlying mechanisms in the otoprotective actions of adenosine. Nevertheless, the detailed discussion of adenosine effects on the cochlear vascular system is over the scope of this review. For more specific coverage of this field please see Vlajkovic et al. 2009 [72], and Munoz et al. 1999 [136].

## 5. Sensorineural Hearing Losses

Among many neurological diseases with increasing prevalence, hearing loss (HL) is of paramount importance affecting more than 400 million people worldwide (https://www.who.int/en/news-room/fact-sheets/detail/deafness-and-hearing-loss, WHO 2018). It could lead to social isolation and depression. Contrary to conductive hearing impairments, SNHLs are a major challenge for pharmaceutical sciences. There are no highly-effective, drug agency-approved specific pharmaceuticals against SNHLs, except for symptomatic approaches with moderate efficacy. The complex, multifactorial pathomechanism of SNHLs and the insufficient knowledge of the basic molecular mechanisms of normal and impaired adult hearing and of the endogenous protective factors hinders drug development. Use of ototoxic medications (Drug-Induced Hearing Loss), overexposure to loud sounds and aging itself (NIHL: Noise-Induced Hearing Loss; AHL: Age-related Hearing Loss) are the most prevailing forms of SNHLs [137].

Numerous pharmacological agents used in the clinical practice have been proven to be ototoxic such as aminoglycoside antibiotics, chemotherapeutic drugs or diuretics. Despite of their side-effects, aminoglycosides are selected for the treatment of sepsis, health-care and nosocomial pneumonias, urinary tract infections and other severe infections caused by gram-negative bacteria due to a number of advantageous properties (e.g., low-level antibacterial resistance, economical cost) [138,139]. Susceptibility to aminoglycoside ototoxicity depends on the route of administration, the genotype and other existing diseases [140]. Bacteriogenic-induced systemic inflammation exacerbates the extent of aminoglycoside-induced hearing loss [141] by enhancing their cochlear uptake [18]. The prevalence of hearing loss resulting from their use varies between 2%–25% across the literature [139]. Aminoglycoside-induced permanent cochlear damage affects mostly OHCs and IHCs [140,142], however, damage of SV, supporting cells and SGNs can also be observed [142]. Main mechanisms of cell injury caused by aminoglycosides are excitotoxicity [143] and increased amounts of reactive oxygen species (ROS) [140,144,145]. Furthermore, inhibition of ATP receptors may also play a role in the development of the disorder [146].

NIHL is the most prevalent form of occupational injuries. Work-related noise exposure contributes to approximately 16% of disabling hearing impairment in adults [137]. Furthermore, as a consequence of change in leisure activities (e.g., listening to MP3 players, headphones, loud concerts), an increasing number of people are suffering from NIHL [137]. Around 1.1 billion teens and young adults (aged between 12–35 years) are potentially in danger in terms of hearing loss due to recreational exposure to loud noises (https://www.who.int/en/news-room/fact-sheets/detail/deafness-and-hearing-loss, WHO 2018). Excitotoxicity [147] and oxidative stress [148,149,150] are presumed to play a role in the progression of NIHL. Moreover, abnormal increase in intracellular Ca^2+^ [151,152], swelling of SV [153,154], noise-induced inflammation [155] and damage of ATP-mediated processes are further significant pathological factors. Intense sound or noise exposure triggers cochlear inflammation by upregulation of pro-inflammatory mediators (e.g., TNF-α, IL-1β and IL-6) [156] and adhesion molecules (e.g., ICAM-1/CD54) [157] presumably leading to the exacerbation of cochlear damage [155]. Cochlear signaling via the P2X2 purinergic receptor has protective function against noise-induced permanent increase in the auditory threshold. The malfunction of this rescue response promotes the development of NIHL [158]. During these harmful processes, auditory hair cells and auditory neurons have been known to be the two principal cell types that fail but recently the recognition of the importance of supporting cells in the organ of Corti has emerged [159,160,161,162,163].

Presbycusis or AHL in the elderly population is the most frequent form of SNHLs with increasing prevalence due to average age increase. Age-related decrease in hearing ability mostly begins in young adulthood (during the late 30s) showing gradual deterioration of hearing over time. Every third person over the age of 65 has a certain degree of hearing loss [137]. Factors underlying the development of this multifactorial disease are cochlear aging, environmental effects (e.g., harmful noise), genetic predisposition and co-existing disorders [137]. Through the years, these factors lead to cumulative damage of the hearing system primarily affecting hair cells, SGNs and the SV [164]. Similarly to aminoglycoside-induced hearing loss and NIHL as mentioned above, excessive release of glutamate from IHCs and disturbance in the balance between the production and degradation of ROS are key mechanisms in AHL [107]. Moreover, inflammation and purinergic signaling are also involved in this pathological condition. The chronic, low-grade stimulation of the immune system-called “inflammaging”—contributes to age-related diseases including AHL [165]. In addition, dysfunction of ATP-gated P2X2 receptor may exacerbate the progression of AHL [42].

## 6. Purinergic Signaling and Sensorineural Hearing Losses

Previously we summarized the basic control functions of the purinergic system in the cochlea, such as regulation of K^+^ recycling and of cochlear amplification or detection of pathologic changes by generating Ca^2+^ waves. Purinergic signaling is sensitive to pathophysiological changes (e.g., due to noise or ischemia) of the extracellular milieu and thereby can promptly react and initiate protective mechanisms. This control function of the purinergic system has been delineated in sound sensitivity of the cochlea and in NIHLs [74,166]. Noise trauma induces various events and purinergic mechanisms are involved in this machinery. Noise exposure induced up- and down regulation of purinergic receptors and also higher levels of ectonucleotidases was observed in response to deleterious noise stimuli [10,44,78,96].

In vivo experiments mostly supported the idea of purinergic protection in noise trauma revealing also some contradictory findings. For instance, endocochlear potential was decreased when ATP was administered in vivo into the endolymph of rodents. It probably resulted in reduced driving force on K^+^ influx activating the hair cells. Non-selective P2 receptor antagonists reversed this protective effect of ATP confirming the involvement of P2 receptors [41,88,167]. Both electrocochleographic (cochlear microphonic, summating potential and compound action potential) and otoacoustic emission (DPOAE-indicative of the OHC-driven cochlear amplification) measurements revealed diminished cochlear sensitivity after application of P2 receptor agonists (as reduced cochlear potentials and suppressed values in distortion product) [168]. Acoustic trauma causes temporary shift of hearing threshold. ATP, administered into the perilymph, promoted the recovery from this state [169]. Activation of P2X2 receptors located on epithelial cells lining the scala media by endolymphatic ATP resulted in reduced hearing sensitivity by the K^+^ shunt conductance [48], while ATP in the perilymph, acting on supporting cells and on OHCs, had similar effect by modifying the cochlear micromechanics [92,129,170]. Somewhat surprisingly perilymphatic administration of P2 receptor antagonists (PPADS and suramin) also resulted in suppression of the cochlear potentials and DPOAE [170,171]. Furthermore, the perilymphatic PPADS and moderately intense sound exposure evoked similar and additive cochlear potential suppression effects [172]. On the other hand, when measured by DPOAE, perilymphatic PPADS was protective against moderately intense sound [173].

Pathological insults such as noise exposure and perfusion of cisplatin into the perilymph resulted in upregulation of adenosine receptors in guinea pigs [174,175]. A1 agonists prevented cisplatin-induced ototoxicity and this preventive effect was reversed by an A1 antagonist. On the contrary, activation of A2A receptors increased the cisplatin-induced threshold changes [176]. A1 agonists also reduced excitotoxicity caused by kainic acid administration into the perilymph. A2A agonists failed to influence it [75]. Elevation of superoxide dismutase, catalase or glutathione peroxidase activity was detected in different ototoxicity models. Noteworthy is that A1 adenosine receptor activation recruits protective mechanism against the increased level of ROS [174,176].

Regulation of CBF by adenosine and/or ATP can be an additional factor resulting in otoprotective effect of the purinergic ligands. Both adenosine and ATP increased the CBF in guinea pigs [136]. Intense noise exposure resulted in decreased CBF accompanied by a drop in the oxygen tension (pO_2_) of the endolymph. It can be a major contribution to the cochlear damage in NIHLs [177,178,179]. Cerebrovascular diseases affecting the SV in the inner ear may have similar effect [180,181]. An A1 adenosine receptor agonist, administered i.p., proved to be protective against cochlear injury induced by transient occlusion of the labyrinthine artery [182].

Adenosine seems to prevent the development of NIHL, and it was proposed to be a promising future target. Chronic administration (two weeks) of caffeine–a non-selective adenosine receptor antagonist–inhibited the spontaneous regeneration of the hearing thresholds in guinea pigs exposed to noise, indicating that adenosine is involved in the regeneration from harmful-stimuli [183]. Genetic deletion of A1 adenosine receptors caused higher threshold shifts in response to a 2h intense noise exposure in mice. Additionally, ABR measurements revealed decreases in amplitudes of the primary auditory neurons. Enhanced loss of OHCs and synapses was also observed. In contrast, deletion of A2A receptors resulted in increased cochlear resistance to the acoustic trauma [74]. Adenosine Amine Congener, a selective A1 receptor agonist, reduced noise- and cisplatin-induced cochlear injury in rodents. In a time window after the exposure of the insults, it can reduce and inhibit the hair cell loss and it was proposed to be effective as a cochlear rescue agent [184,185]. After i.v. administration, it could reach therapeutic concentration in the cochlea in rats [186]. However, cardiovascular adverse effects (e.g., vasodilation or heart blocks) of direct adenosine receptor ligands may limit their therapeutic use. A tissue-specific intervention at the intracellular level of adenosine receptor signaling could bypass this handicap [187]. Bogosanovich from the Thorne lab at the University of Auckland (Mastersthesis, http://hdl.handle.net/2292/27523) reported promising results when tested CCG-4986, an inhibitor of RGS4 (regulator of G-protein signaling 4). Namely, it proved to be protective against acoustic trauma. When G-protein coupled receptors (such as A1 adenosine receptors) are activated, RGS proteins limit and shorten the response. As the RGS4 subtype shows tissue-specific expression, including cochlear localizations (spiral ganglion neurons, supporting and sensory cells), the inhibitor of this subtype provides a tool to disinhibit (i.e., enhance) the action of endogenous adenosine on A1 receptor in the cochlea without risking systemic adverse effects of adenosine on the cardiovascular system.

However, drawing a clear conclusion about the effects of ATP and/or adenosine on cochlear functions especially in pathophysiological conditions is challenging. Predominantly otoprotection seems to be the primary effect of the P1 and/or P2 receptor agonists. However, the effect of a purinergic ligand can depend on (i) its concentration and way of administration (peri- or endolymphatic), (ii) the intensity of the noise exposure (iii) the experimental method used to assess the function of hearing and the ATP effect is mediated by (iv) the diversity of the purinergic receptor subtypes located at different sites in the cochlea.

Besides the effects on mechanotransduction and on cochlear adaptation mechanisms, purinergic mechanisms may have an additional impact on cochlear functions by influencing the CBF. A third peculiar factor in the scenario how the purinergic system is involved in cochlear pathophysiology can be the immune system. In spite of the enormous enthusiasm to clarify the purinergic mechanisms influencing the immune system (it will be briefly discussed in the next chapter), less attention was paid to the purine-immune interactions in the inner ear. Here we propose that the understanding of the purinergic effects in case of cochlear damage requires the substantial discussion and elucidation of this factor.

## 7. Purinergic Signaling and the Immune System—Possible Impact on Sensorineural Hearing Loss

As we mentioned above, BLB doesn’t isolate the cochlea completely from the systemic immune system: resident macrophages are present in the organ of Corti, also in physiologic conditions. Injury (e.g., noise exposure, ischemia) evokes an immune response with cytokine release and invasion of immune cells from the periphery [15,16,17,18,19,20].

Purinergic signaling is fundamentally involved in the coordination of immune responses, e.g., against invading pathogens. However, inadequate purinergic signaling may be resulted in excessive inflammation and further pathological events in chronic inflammatory diseases [188]. Recent comprehensive reviews characterize the purinergic mechanisms influencing the functioning of the immune system, for further reading please see, e.g., Burnstock and Boeynaems 2014 [189], Cekic and Linden 2016 [190], Haskó and Cronstein 2013 [191], Linden et al., 2019 [192].

Here we summarize briefly the present knowledge on this peculiar field, relevant to cochlear functions. Most immune cells such as leukocytes (neutrophils, eosinophils, basophils, and mast cells), monocytes, macrophages/microglia, dendritic cells, lymphocytes and natural killer cells express multiple purinergic receptors [193]. P2X7 and P2Y2 receptors seem to be the major purinergic receptor subtypes but other purinergic subtypes such as A2A adenosine as well as P2Y12 and P2Y1 receptors are also involved in immune functions and inflammation [192,194,195,196,197,198,199].

P2X7 receptors are expressed predominantly in immune cells, in virtually all immune cell types, and they are considered as pro-inflammatory receptors. A unique feature of this receptor, namely it requires much higher ATP concentrations (>100 µM) for activation than other P2X channels, has a significant impact on its peculiar character. Agonist binding resulted in opening of the channel and influx of small cations (Ca^2+^ and Na^+^). Prolonged activation is accompanied (within seconds) with opening of a large pore allowing the passage of molecules with a mass up to 900 Da [200]. It can be resulted in a massive disturbance of cytoplasmic ion homeostasis [199]. Furthermore, P2X7 receptors activate transcription factors such as NF-κB [201] and NFAT (Nuclear Factor of Activated T-cells) [202], and they are in central position of inflammatory events. Activation of the inflammasome is accompanied by increased production of several pro-inflammatory cytokines (e.g., IL-1β and TNFα), stimulation of early inflammatory gene expression and increased T cell proliferation [203]. P2X7 receptor is linked to possible cytotoxic effects; it can be crucially involved in pathological inflammatory events, apoptosis and cell death [204].

P2Y2 receptors are localized mostly in leukocytes (e.g., neutrophils, eosinophils, dendritic cells) and macrophages in the immune system. It is substantially involved in classical leukocyte functions such as mediator production and migration [188]. ATP released from damaged or apoptotic cells acting on P2Y2 receptors evokes a chemotactic find-me signal resulting in attraction and migration of immune cells and their accumulation at the site of inflammation [205]. Thereby P2Y2 receptor activation contributes to clearing of apoptotic cells [206]. It can also be involved in release of cytokines such as IL-8 [207]. P2Y2 receptor is proposed to play a protective role in the immune system, as it activates defence mechanisms such as clearance of damaged cells. However, it can be involved in overactivation and pathologic alterations of the immune mechanisms resulted in chronic inflammatory disease states and fibrotic remodelling [188].

P2Y12 is expressed on microglial cells, where its activation induces chemotaxis in response to tissue injury [208,209]. It is supposed to have a key role in the regulation of microglial responses to activatory signals [210]. Antigen endocytosis of dendritic cells is also mediated by P2Y12 receptors [211]. P2Y12 receptor activation may result in both pro-inflammatory and protective events, thereby improving or worsening the situation [194,212,213]. 

One of the major locations of P2Y1 receptors is the central nervous system, where it is expressed in both neurons and glial cell types [25,214]. Its well documented function is neuromodulation [194,215,216,217]. In astrocytes P2Y1 receptor activation can stimulate the release of IL-6 and it can be involved in astrocyte-mediated neuroprotection [218]. It is also implicated in stimulation and migration of microglia [219].

Further P2 receptors are also involved in immune functions such as P2X4 (microglial activation and migration, activation of T cells, regulation of autophagy in macrophages), P2Y6 (chemotaxis and microglial phagocytosis), P2Y11 (maturation, migration and regulation of cytokine release in dendritic cells, regulation of chemotaxis in natural killer cells) as well as P2Y13 and 14 (degranulation of mast cells) [189].

Adenosine is considered as an endogenous anti-inflammatory agent [191]. Adenosine has inhibitory effects on neutrophils: mediated mainly by A2A and A2B receptors [191,220,221]. A3 receptor activation can be synergistic with P2Y2 to amplify the migration of neutrophils, A2A receptors may exert an inhibitory effect on this function [205,222]. Activation of A3 receptors is resulted in increased mast cell degranulation, while inhibition of degranulation was observed by A2A and A2B receptors [189,223]. A2A and A2B receptor activation resulted in mostly inhibitory effects on monocytes and macrophages [224]. A1 and A3 receptors stimulated microglial functions (e.g., migration), in contrast the A2A receptors may be inhibitory on microglial functions [189,225]. Adenosine, activating A2A receptors exerts mostly inhibitory effects on T cells [226].

In contrast, conflicting data has been revealed regarding the effects of adenosine in neuroinflammation in the central nervous system [227,228]: adenosine in high concentration caused (mostly A2A receptor mediated) pro-inflammatory effects [229], such as inflammasome activation, IL-1β production, recruitment and activation of microglia and alterations in astrocyte function [230,231]. High concentration of glutamate observed in brain injury has been proposed to switch the effect of A2A receptor activation from anti-inflammatory to pro-inflammatory in case of brain injury and neuroinflammation [228]. This phenomenon highlights the complexity of purinergic signaling in the immune system and also of the possible therapeutic utilization of ligands targeting the purinergic system.

As we described above, the purinergic adaptation ensures protection of the hearing system in physiological conditions and in response to transient deleterious insults. However, disturbances of tightly controlled and balanced systems such as the regulation of the hearing sensitivity might quickly result in pathologic changes. When a deleterious insult persists, extracellular purines can reach pathologically high concentrations. Under these circumstances the purinergic mechanisms can reveal their Janus-faced character: Fine-tuned and well-controlled actions in physiological conditions, overactivation and cytotoxicity in a severe pathologic scenario (with the dominant involvement of e.g., the P2X7 receptors).

We pointed out, that elements of the immune system are present in the cochlea. Activation of the immune system is necessary to eliminate dangerous signals but overactivation can be deleterious. Some elements of the purinergic modulation can be involved in these “lost-of-balance” events. Especially P2X7 receptor can be a candidate to be involved in such immune-mediated deleterious events. Therefore, here we propose to contemplate immune-mediated effects in the horizon of possible novel purinergic targets in the therapy of sensorineural hearing disturbances. The complexity of purinergic signaling in the organ of Corti and some contradictory reports related to this field can be better understood when the purinergic modulation of immune mechanisms in cochlear pathologies will be kept in focus.

## 8. Immune Mechanisms in Sensorineural Hearing Losses

Inflammatory reactions and immune responses are there not exclusively to protect against invading pathogens but to maintain body homeostasis and restore tissue function after acute stress like sterile mechanical or chemical injury or even physiological apoptotic cell death [14]. Debris of dead cells forming damage-associated molecular patterns (DAMPs) binds to pattern recognition receptors (PRRs) on macrophages and activates the immune response [232,233,234]. Recent results suggest a similar sterile inflammation and immune system function in the cochlea [232,233,234], which has been considered before as an immune response-free area separated from the systemic circulation by the BLB. Even the ageing of the cochlea involves immune reactions in a chronic form [13,20,235].

Inflammation and the immune response itself is there to protect the cochlea, but its toolkit can easily turn against its own host and can also contribute to the pathomechanism of the different SNHLs. Inhibition of these self-destructive mechanisms or termination of the immune response in time before it turns to self-destructive are feasible therapeutical approaches [14].

### 8.1. Components of the Cochlear Immune System

Cell types necessary to accomplish the immune response can be found in the hearing organ [236,237].

Resident macrophages are the constitutive immune cells of the cochlea. They are distributed in the spiral limbus, spiral ganglion, along the peripheral processes of the SGNs, spiral ligament, SV and occasionally on the scala tympani side of the BM [15,16,20,238,239,240,241,242] (Figure 1). The resident macrophages are activated by different types of insults like acoustic overstimulation, ischemia, cytotoxic agents or local surgical stress [15,16,17].

Damaging factors also induce the infiltration of monocytes from the blood. These cells of hematopoietic origin join the resident macrophages and differentiate into monocyte-derived macrophages [242].

Although the vast majority of immune cells in the cochlea are macrophages, granulocytes, T and B lymphocytes and natural killer cells were also identified in very low proportion (some percent), at least in the postnatal stage [243].

The scala media is free of professional phagocytic immune cells probably to avoid any self-attack on the unique sensory epithelium. Necessary scavenging of apoptotic/necrotic sensory cells in the organ of Corti can be performed by supporting cells that transdifferentiate to non-professional phagocytes triggered by hair cell damage and death [20,21].

Fibrocytes in the spiral limbus, spiral ligament and SV are also involved in cochlear inflammation and innate immune responses [15,155,156,237,244,245,246].

The perivascular-resident macrophage-like melanocytes in the SV contributes to the BLB and their genetically induced depletion results in a substantial drop in endocochlear potential and hearing loss [16,241].

### 8.2. Activation, Resolution and Function of the Cochlear Immune System 

Different cochlear insults and cellular damages activate resident macrophages in the cochlea and evoke the infiltration of monocytes [15,247,248]. This is contrary to other organs and tissues where recruitment of neutrophil granulocytes usually precedes monocyte infiltration [249,250]. In the cochlea, cells of the monocyte-macrophage lineage are the major immune cell types [251]. Activated resident and newly differentiated macrophages express pro-inflammatory mediators like TNF-α, IL-1β, IL-6, leucotrienes, tromboxanes, PGD2 or the pro-inflammatory chemokines such as CCL2, CCL4 and CXCL12 (chemokine ligand 2 and 4, C-X-C motif chemokine 12) [14,155,156,157,158,237,248,252,253]. Among these, TNF-α seems to be a primary and essential one participating in the initiation, amplification and maintenance of inflammation in the cochlea [254,255,256]. At the peak of inflammation, when its homeostatic role has been largely completed, the release of pro-inflammatory mediators start to decrease simultaneously with the elevation of anti-inflammatory ones (e.g., IL-10, TGF-β, PGE2). In the meantime, synthesis and release of pro-resolution mediators, like lipoxins, resolvins, protectins, maresins and annexin A1 (ANXA1) are initiated. Pro-resolving mediators switch off expression and signaling of pro-inflammatory mediators plus boost the apoptosis of inflammation-recruited and transformed immune cells and the clearance of damaged tissue [14]. ANXA1 [257,258,259], which can be found in different cell types of the SM, especially in lipid droplets of the HCs has a crucial role in the regulation of cochlear inflammation [14,260]. Its G protein coupled ALX/FPR2 receptors (formyl peptide receptor 2) are abundant on IHCs, OHCs, DCs and pillar cells and also expressed in cells lining the scala tympani and vestibuli [260]. In addition to inhibiting pro-inflammatory cytokines and detrimental monocyte infiltration of the scala media, ANXA1 promotes the expression of other pro-resolving mediators and stimulates organ of Corti supporting cell transformation to non-professional macrophages and the reprogramming of macrophages toward resolving phenotype to provide phagocytes for tissue-repair [260,261]. Furthermore, prominent role of ANXA1 is substantiated by the protective role of glucocorticoids in SNHLs. Stimulation of the synthesis and release of ANXA1 is playing a prominent role in the anti-inflammatory action of these corticosteroids [260,262,263,264].

Recognition of necrotic or apoptotic hair cells and non-receptorial cells of the organ of Corti is based on their liberated intracellular components which serves as DAMPs or advanced glycation endproducts (AGEs) and are recognized by PRRs. The toll-like receptors (TLRs) and the receptor for advanced glycation endproduct (RAGE) bind their respective ligands and activate NF-kB, which induces the transcription of inflammatory cytokine genes [158,233,234,265]. The release of pro-inflammatory cytokines, chemokines and ROS from macrophages induced by their PRR activation in turn recruit further blood-derived monocyte that differentiate into macrophages capable to release inflammatory mediators, too [253]. Over cytokine and other inflammatory mediator production, macrophages also phagocytose cochlear cells and their debris.

Different types of resident, non-professional immune cells are also involved in the activation and duty of inflammation and immune response. Supporting cells, transformed to and working as non-professional phagocytes [21] clear up injured and apoptotic hair cells and express many immune- and inflammation-related molecules [266]. Cochlear fibrocytes supposed to play a similar role. They activate inflammation and the immune response [15,244,245,246] by releasing pro-inflammatory cytokines (TNF-α, IL-1β and IL-6) [155,156,237,266] and damage mediators like ROS [156,265,267] and adhesion molecules [155,237,267] upon cochlear stress, while their stress-induced damage recruit CD45(+) bone marrow-derived white blood cells [15], essentially monocytes.

Infiltrating monocytes and pro-inflammatory cytokines together with other damage mediators such as ROS destroy cochlear cells [158,268]. Suppression of the immune response by blocking cytokine signaling mitigates hair cell damage [158,269]. On the contrary, resident macrophages in the cochlea can activate pro-survival signaling [107,270]. Fractalkine receptor (CX3CR1) expressing macrophages provided protection of hair cells [269]. The association of inflammation and immune response with both cochlear damage and protection and regeneration [253] is in accordance with the well-known Janus face of the immune system in the body.

### 8.3. Role of the Cochlear Immune System in SNHLs

The original idea about the role of inflammation in SNHLs came from the otoprotective effect of glucocorticoids, that are strong immune suppressants [271].

Inflammation and immune response are involved in the pathomechanism of many different forms of SNHLs. Excessive noise [155,156,157,237,248,252,272], ototoxic aminoglycoside antibiotics or cisplatin [256,269,273,274,275,276], aging [13,165,235,277,278,279], cochlear surgery and implantation [242,280,281] or hair cell ablation [238] all activate inflammation and immune response that are characterized by a massive infiltration of inflammatory cells into the cochlea. These studies also showed a correlation between the inflammation and the damage of the cochlea and impairment of hearing [14]. The activated resident macrophages and invading monocytes release pro-inflammatory cytokines, chemokines and ROS which can both damage cochlear tissue and recruit more immune competent cells by promoting further infiltration [253]. The mechanism of tissue damage performed by inflammation also involves the up and down regulation of the expression of microRNAs as it was shown in different forms of SNHLs such as NIHL, AHL, Sudden or Nonsyndromic SNHLs [282]. Besides damage in the organ of Corti, the inflammation is also associated to SGN injury [238,283].

On the other side, the immunosuppressive phenotype of blood-borne macrophages (denoted M2 in other tissues) [240] and the macrophages transdifferentiated from supporting cells of the organ of Corti [20,21] have a prominent role in phagocytic disposal of damaged or dead hair cells and any cell debris and in tissue repair, including scar formation [21] in the organ of Corti or auditory nerve regeneration [284]. These macrophages secrete anti-inflammatory cytokines and neurotrophic factors [240].

In this balanced, activated-then-resolved form the immune system is a normal and indispensable adaptive process, which ensures the normal structure and function of the cochlea. Either the overactivation of the immune response or its delayed, incomplete resolution contribute to the pathophysiology of SNHLs [14,107,240,270].

The complex regulation of the immune system requires continuous communication between its components. Purinergic signaling is a prominent applicant for this purpose. Hair cell death alone recruits macrophages and the liberated intracellular ATP is a presumed death-reporting signal [111] that activates purinergic receptors on leukocytes [285]. The protection and neurotrophic stimulation of the auditory nerve exerted by resident macrophages is suggested to be dependent on extracellular adenosine [240].

It seems that tonotopy, fundamental feature of the cochlea, appears in the activation of the immune response in SNHLs. Apical and basal turn macrophages shows different phenotypes and response patterns to sensory cell degeneration in AHL [13] and only the basal macrophages display marked activation of antigen-presenting function for acoustic trauma [248].

### 8.4. Role of the Cochlear Immune System in NIHLs

Traumatic noise exposure-evoked inflammatory response in the cochlea involves activation of resident macrophages [17], upregulation of pro-inflammatory mediators and rapid recruitment of inflammatory cells from the vascular system [15,17,156,157,252,286]. Secretion of the pro-inflammatory cytokines (TNF-α, IL-1β and IL-6) [156,252], chemokines (e.g., MCP-1/CCL2) [157], ROS [265,267] and damaging adhesion molecules [244,267] is enhanced by NF-κB, which is upregulated by the acoustic overstimulation [155,244,265,267]. Cytokines, such as the TNF-α are well known damage mediators in the cochlea [268].

Excessive noise also evokes cochlear ischemia, which increases the expression of pro-inflammatory TNF-α and supress the protective IGF1 through the stabilization of the transcription factor HIF-1a (hypoxia-inducible factor 1a) [287].

Infiltration of immune cells appears fairly quickly. Within hours after acoustic overstimulation leukocytes from the lateral wall and the spiral limbus migrate to the scala vestibuli and scala tympani [15,157,252,288,289], the scala vestibuli side of the RM [288] and the scala tympani side of the BM [157,248]. 

The kinetics of the release of pro-inflammatory mediators show a double peak profile with an early upregulation at 6 h and a second peak at seven days after noise exposure. The first peak is suggested to be related to the recruitment of inflammatory cells into the cochlea while the second one is probably associated with reparative processes [237]. Another explanation attributes the first peak to the resident macrophages and fibrocytes and the second to the infiltrating immune cells [253].

Fibrocytes of the spiral ligament express inflammatory cytokines as a result of acoustic overstimulation [155,156,237] and acoustic trauma results in considerable damage in fibrocytes in the spiral ligament and spiral limbus [15].

Chronic, moderate noise exposure also activates an inflammatory response with a two weeks plateau, suggesting the participation of the immune response in the pathophysiology of this chronic form of NIHL, as well [237].

### 8.5. Role of the Cochlear Immune System in Aminoglycoside- and Cisplatin-Induced Hearing Losses

Aminoglycoside antibiotics and cisplatin activate inflammatory cells and enhance the expression of inflammatory mediators either directly or indirectly through ROS generation and this immune response contributes to the hearing loss they evoke [275,276]. Similarly to NIHL-related immunological responses, increased number of macrophages appear in the spiral ganglion, the lateral wall and the habenular region after aminoglycoside administration [269,290,291,292]. These macrophages promote hair cell death by inducing the release of cytotoxic cytokines [270]. Cisplatin increases the expression of inducible nitric oxide synthetase (iNOS), cyclooxygenase 2 (COX-2) and TNF-α through direct activation of nicotinamide adenine dinucleotide phosphate oxidase 3 (NOX3). Activated NOX3 induces ROS generation that leads to the phosphorylation of STAT-1 in OHCs, SG cells and regions such as SV and spiral ligament. Activation of STAT-1, a transcription factor regulating inflammatory mediators, results in the apoptosis of OHCs [275]. Cisplatin increases the level of IL-6, IL-1β and TNF-α in HEI-OC1 cells and in several parts of the cochlea (OHC layers, spiral ligament, spiral limbus and SV) through the activation of ERK and NF-kB [256]. These pro-inflammatory cytokines, predominantly TNF-α, contribute to the sensory hair cell damage in the inner ear [256]. Histamine and serotonin, produced in a pre-existing inflammation increase the permeability of the BLB to aminoglycosides thus cause the exacerbation of the drug-induced hearing loss [18,141,293].

### 8.6. Role of the Cochlear Immune System in AHLs

Age-dependent chronic, low-level activation of the immune system (“inflammaging”) is a key factor in AHL [165,235]. Low-grade inflammation may be also linked to some of the auditory problems usually associated with aging [235]. In the aging cochlea, degeneration of sensory cells and SGNs provoke the cochlear inflammatory response [13,20]. It seems that resident macrophages, not recently infiltrated monocytes, play an active role in the age-dependent degeneration of hair cells by contributing to deterioration of the sensory cell microenvironment. These macrophages, on the scala tympani side of the BM display dynamic changes in number and morphology as age increases and they also show amoeboid transformation in regions where sensory cell degeneration has not yet initiated [13]. Failing immuno-competent cells may also contribute to some types of accelerated AHL [277].

### 8.7. Opposing Harmful Cochlear Inflammation by the Purinergic System–Therapeutic Potential

Inhibition of the harmful immune responses and promotion of the resolution of inflammation in time are two possibilities to cure SNHLs that involves inflammatory components in their pathomechanisms. A potential way of this approach is the modulation of the purinergic system, which influences almost all aspects of the immune response [189,190,191,192]. Termination of the immune response in the appropriate time is part of a normal inflammatory process in the cochlea [14]. Several specific pro-resolving mediators are part of this task including the purines [191,294,295]. Tan et al. raised the possibility of treating NIHL by mitigating noise-induced cochlear inflammation [155]. Activation of A1 adenosine receptors by its agonist R-phenylisopropyladenosine provided protection against cisplatin-induced ototoxicity by supressing the NOX3/STAT1-mediated inflammation [71].

## 9. Conclusions and Outlook

Based on the involvement of both the purinergic signaling and the immune system in cochlear pathologies as well as the role of the purinergic system in the modulation of immune mechanisms, purinergic targets in immune-mediated effects in the cochlea might be novel promising pharmacotherapeutic possibilities in sensorineural hearing disturbances. 

Unfortunately, the enthusiasm in research aiming to clarify P2 receptor mediated mechanisms and to explore purinergic pharmacotherapeutic targets in the cochlea were reduced in the last few years-no new in vivo result was published. New impulses are needed to conceive this research field. Here we propose the elaboration of purine research on cochlear functions: Investigation of purine-immune interaction in cochlear damage and toxicity. 

## Figures and Tables

**Table 1:** Expression of P2 purinergic receptors in the cochlea before and after hearing onset (P15) in rodents. It includes only subtype and cell specific results. mRNA detection means PCR analysis and in situ hybridization, protein detection means Western-blot analysis and immunohistochemistry, while functional detection indicates pharmacological analysis in electrophysiological or Ca^2+^ imaging experiments. Results from guinea pigs are not separated by age as these animals have in utero hearing. These data are shown in the second part (P > 15) of the table. IHC: Inner hair cell; OHC: Outer hair cell; SGN: Spiral ganglion neuron; IBC: Inner boarder cells; IPhC: Inner phalangeal cell; PC: Pillar cell; DC: Deiters’ cell; HC: Hensen’s cell; OSC: Outer sulcus cell; SV: Stria vascularis; RM: Reissner’s membrane.

**P2 Receptor Subtypes**			**Presence in the Cochlea (P < 15)**									
	**Species**	**Detected**	**OHC**	**IHC**	**SGN**	**IBC & IPhC**	**PC**	**DC**	**HC**	**OSC**	**SV**	**RM**
P2X1	rat	protein			[61]							[61]
P2X2	rat	mRNA	[47,52]	[47,52]	[47,52,56,57]	[47]	[47]	[47,52]	[47]	[47]	[47]	[47]
	mouse	protein	[42,49]					[49]	[49]			[49]
		function	[46]									
P2X3	rat	mRNA			[56,57]							
		protein			[60]							
	mouse	protein	[65]	[65]	[65]							
P2X4	rat	mRNA			[56,57]							
P2X7	rat	mRNA			[56]							
		protein						[62]				
P2Y1	rat	protein										[66]
P2Y2	rat	protein	[66]	[66]			[66]	[66]	[66]	[66]		
		function							[67]	[67]		
P2Y4	rat	protein	[66,68]	[66,68]	[66,68]						[68]	[66]
		function							[67]	[67]		
P2Y6	rat	protein	[66,68]	[66,68]	[66]		[68]				[68]	[66]
P2Y12	rat	protein	[66]		[66]		[66]					[66]
P2Y14	rat	protein	[68]	[68]	[68]					[68]		
**P2 receptor subtypes**			**Presence in the cochlea (P > 15)**									
	**Species**	**Detected**	**OHC**	**IHC**	**SGN**	**IBC & IPhC**	**PC**	**DC**	**HC**	**OSC**	**SV**	**RM**
P2X1	rat	protein			[58]							
	guinea pig	protein	[40,44]									
P2X2	rat	mRNA	[10,47]	[10,47]	[10,47,56]	[47]	[10,47]	[10,47]	[47]	[47]	[10]	[10,47]
		protein	[50]		[10,58,59]		[10]	[10,50,59]	[50]	[50]	[58]	[10]
	mouse	protein	[41,48]	[48]	[41]	[48]	[41,48]	[41,48]	[48]	[48]		[41,48]
	guinea pig	mRNA	[51]	[51]		[51]	[51]	[51]	[51]			
		protein	[37,40,43,44]	[37,45]			[37]	[37]	[37]			
		function	[53]	[45,53]	[53]			[54]				
P2X3	rat	mRNA			[56]							
		protein			[58]							
P2X4	rat	mRNA			[56]							
		protein			[58]							
	guinea pig	protein	[40,44]									
P2X7	rat	mRNA			[56]							
		protein		[62]	[62]		[62]	[62]				
	guinea pig	protein	[40,43,44]									
		function					[55]	[55]	[55]	[55]		
P2Y1	rat	protein	[66]							[66]		[66]
	guinea pig	protein	[40,44]									
P2Y2	rat	protein					[66]			[66]		
	guinea pig	protein	[40,44]									
P2Y4	rat	protein	[66,68]	[66,68]	[66,68]		[66]					[66]
		function									[69]	
	guinea pig	protein	[40,44]					[70]	[70]			
P2Y6	rat	protein	[68]		[66,68]		[66,68]				[66]	[66]
P2Y12	rat	protein			[66]							[66]
P2Y14	rat	protein			[68]					[68]		

**Table 2:** Expression of adenosine receptors in the cochlea after hearing onset in rodents. It includes only subtype and cell specific results. mRNA detection means PCR analysis and in situ hybridization, protein detection means Western-blot analysis and immunohistochemistry, while functional detection indicates pharmacological analysis in electrophysiological or Ca^2+^ imaging experiments. In case of mice and rats, results come from animals age P > 15. Results from guinea pigs are not separated by age as these animals have in utero hearing. IHC: Inner hair cell; OHC: Outer hair cell; SGN: Spiral ganglion neuron; IBC: Inner boarder cells; IPhC: Inner phalangeal cell; PC: Pillar cell; DC: Deiters’ cell; HC: Hensen’s cell; OSC: Outer sulcus cell; SV: Stria vascularis; RM: Reissner’s membrane.

Receptor Subtypes			Presence in the Cochlea (P > 15)									
	Species	Detected	OHC	IHC	SGN	IBC & IPhC	PC	DC	HC	OSC	SV	RM
A1	rat	mRNA	[71]	[71]								
		protein	[71]	[71,72,73]	[72,73]			[72,73]				
		function	[71]	[71]								
	mouse	function		[74]	[74]							
	guinea pig	function	[75]	[75]	[75]							
A2A	rat	protein		[72,73]	[72,73]			[72,73]				
	mouse	protein			[76]						[76]	
		function		[74]	[74]							
A3	rat	protein	[72,73]	[72,73]	[72,73]		[73]	[72,73]	[72,73]	[72,73]		

**Table 3:** Expression of ectonucleotidases in the cochlea before and after hearing onset (P15) in rodents. It includes only subtype and cell specific results. mRNA detection means PCR analysis and in situ hybridization, protein detection means Western-blot analysis and immunohistochemistry, while functional detection indicates pharmacological analysis in electrophysiological or Ca^2+^ imaging experiments. NTPDases: Ectonucleoside triphosphate diphosphohydrolase isoenzymes; IHC: Inner hair cell; OHC: Outer hair cell; SGN: Spiral ganglion neuron; IBC: Inner boarder cell; IPhC: Inner phalangeal cell; PC: Pillar cell; DC: Deiters’ cell; HC: Hensen’s cell; OSC: Outer sulcus cell; SV: Stria vascularis; RM: Reissner’s membrane.

**Ectonucleotidases**			**Presence in the Cochlea (P < 15)**									
	**Species**	**Detected**	**OHC**	**IHC**	**SGN**	**IBC & IPhC**	**PC**	**DC**	**HC**	**OSC**	**SV**	**RM**
**NTPDase5**	rat	protein	[68]	[68]	[68]	[68]	[68]	[68]	[68]	[68]	[68]	
**NTPDase6**	rat	protein	[68]	[68]								
**Ectonucleotidases**			**Presence in the cochlea (P > 15)**									
**NTPDase1**	rat	protein	[77]	[77]	[77]		[77]	[77]		[77]	[78]	
	mouse	protein			[78,79,80]						[79,80]	
**NTPDase2**	rat	protein	[77]	[77]			[77]				[77,78]	
	mouse	protein	[80]	[80]	[78]			[80]				
**NTPDase3**	rat	protein	[81]	[81]	[81]		[81]				[81]	
**NTPDase5**	rat	protein			[68,82]	[82]	[68]	[68,82]				
**NTPDase6**	rat	protein		[68,82]								

## Abbreviations

ABR	auditory brainstem response
AHL	age-related hearing loss
ATP	adenosine triphosphate
BLB	blood-labyrinth barrier
CBF	cochlear blood flow
COX-2	cyclooxygenase 2
DAMP	damage-associated molecular pattern
DC	Deiters’ cell
DPOAE	distortion product of otoacoustic emission
HC	Hensen’s cell
IL-1β	interleukin 1 beta
IL-6	interleukin 6
iNOS	inducible nitric oxide synthetase
NFAT	nuclear factor of activated T-cells
NF-κB	nuclear factor kappa-light-chain-enhancer of activated B cells
NIHL	noise-induced hearing loss
NOX3	nicotinamide adenine dinucleotide phosphate oxidase 3
OHC	outer hair cell
PPADS	pyridoxalphosphate-6-azophenyl-2’,4’-disulfonic acid
PRR	pattern recognition receptor
RGS	regulators of G protein signaling
RGS4	regulator of G-protein signaling 4
ROS	reactive oxygen species
SGN	spiral ganglion neuron
SNHL	sensorineural hearing loss
STAT-1	signal transducer and activator of transcription 1
SV	stria vascularis
TNFα	tumor necrosis factor alpha
